# Solidarity tourism: A pathway to revitalising the health of vulnerable war-affected populations?

**DOI:** 10.7189/jogh.13.03050

**Published:** 2023-09-01

**Authors:** Jun Wen, Fangli Hu, Danni Zheng, Ian Phau, Metin Kozak, Haifeng Hou, Wei Wang

**Affiliations:** 1 School of Business and Law, Edith Cowan University, Joondalup, Australia; 2 Centre for Precision Health, Edith Cowan University, Joondalup, Australia; 3 Department of Tourism, Fudan University, Shanghai, China; 4 School of Marketing, Curtin Business School, Curtin University, Bentley, Australia; 5 Department of Advertising, School of Communication, Kadir Has University, Istanbul, Turkey; 6 School of Public Health, Shandong First Medical University and Shandong Academy of Medical Sciences, Jinan, China; 7 The Second Affiliated Hospital of Shandong First Medical University, Taian, China

## WAR, TOURISM, AND GLOBAL HEALTH: AN INTERDISCIPLINARY VIEW

The struggles of war are felt by all who occupy an affected region (and beyond), irrespective of whether they are in active combat. This experience has physical effects (e.g. injury, illness, malnutrition, disability, sexual violence, and/or death) and emotional impacts (e.g. posttraumatic stress disorder, depression, and anxiety) [1-3]. The terror associated with war disrupts lives and relationships, leaving individuals, families, and communities distressed. Due to such immediate and long-term adverse outcomes, war represents a highly destructive and enduring public health emergency [4]. Unfortunately, war has persisted into the 21st century, from the Second Congo War (1998-2003) to the Sino-Indian Border Dispute (2020) [5]. As people continued to grapple with the stresses of coronavirus disease 2019 (COVID-19) in 2022, the Russia-Ukraine conflict was reignited [6] for the first time since November 2013 [7]. This war has captured the world’s attention and remains front and centre on international news and social media. It has brought with it catastrophic health and humanitarian crises that are only compounded by COVID-19. As of 13 February 2023, 18 955 Ukrainian civilian casualties (7199 killed and 11 756 injured) have been documented from this conflict [8]. Ensuing environmental pollution and infrastructure disruption may pose persistent and even intergenerational health hazards to locals [9]. Thankfully, recent reports on the Russia-Ukraine conflict have suggested that many industries, including tourism, have begun to offer financial and psychological support to people living in war zones. For instance, Airbnb introduced a financial support campaign by opening temporary housing for Ukraine refugees [10]. International authorities, organisations, and volunteers have been actively providing humanitarian aid and psychosocial support for millions of displaced people.

Among various other global industries, tourism can be a vital force contributing to cultural understanding and world harmony. It can also cultivate a peaceful atmosphere in affected communities and a unique pathway to enhance global health [11-13]. Authorities, scholars, and practitioners should reframe the virtues of tourism as ways to enhance communication, understanding, and tolerance between cultures and nations [12]. In recent decades, wars in Afghanistan, Iraq, Syria, and Yemen destroyed natural and cultural heritage and devastated the nations’ tourism industries and public health [14]. Perceptions of terror and war may cast a shadow on a geographic scale that extends well beyond combat zones’ borders. Thus, exploring how to battle stereotypes and inspire mutual values among humankind may become the main mission in restoring public health and destination images in war-torn areas. People’s shared tourism experiences and post-war tourism activities could even foster optimism, hope, happiness, and resilience – all of which may benefit the mental health and emotional status of those living in war zones.

In response to an emerging call to discuss ways to help Ukraine and other war-torn countries, this paper offers interdisciplinary contemplation about how solidarity tourism can benefit people living in war zones and possibly revitalise global health. In the context of war or military conflict, solidarity tourism refers to “action taken by governments, tourism businesses and tourists to help people suffering during and after crises, driven by empathy towards people, a sense of unity, and a shared understanding of societal standards and responsibilities” [15]. Taking the 2022 Russia-Ukraine conflict as an example, it is imperative to propose ways to demonstrate tourism’s power: from catalysing peace and justice [16], improving mental health [17], and advocating for human rights [18] to furnishing emotional support [19]. Solidarity tourism can be practised via two pathways. One is to encourage people to travel to war zones when it is safe to do so, such as through volunteer travel, group tours, or independent trips [15]. In addition to nature or culture-based tourism, dark tourism (i.e. travel to sites of death, tragedy, and suffering) is a feasible post-war tourism mode with several potential benefits. It offers chances for education, remembrance, and reflection on historical events and their societal impacts; these opportunities foster empathy and a sense of collective memory [20,21]. Dark tourism can also revitalise local economies by attracting visitors and generating revenue. This economic boost supports local infrastructure, conservation efforts, and job creation, ultimately enhancing community well-being [22]. War-affected populations’ mental health may also improve thanks to cross-cultural interaction [23]. However, because dark tourism can be seen as exploiting others’ misfortune, responsible tourism practices should be prioritised to mitigate community or personal harm [24]. Travelling to war-worn areas carries risks of physical injury and emotional challenges. One’s safety can be maximised with proper pre-trip preparation: familiarising oneself with the destination (e.g. potential risks), obtaining travel insurance that covers medical emergencies, and bringing essential safety equipment [25]. Staying informed can further ensure safe travel [26]. Mental burdens can be minimised by travelling with companions or a professional guide [27].

The second pathway involves organising trips for people affected by war (e.g. children, refugees). Yet removing individuals from war-torn areas for a vacation and later returning them to these traumatic environments may raise concerns such as re-traumatisation, unrealistic expectations, and a lack of long-term solutions. It is essential to weigh risks and benefits and address any concerns appropriately [28,29]. Suitable trips enable residents to leave their devastated home areas. This escape gives people temporary respite from the trauma of war along with much-needed psychological assistance [30]. Some trips can expose vulnerable individuals to new cultures, food, environments, and social interaction – possibly inspiring hope while reducing mental distress [31]. Such experiences may also grant war-affected individuals access to advanced medical facilities and health services to heal their wounds [32]. Holiday plans must be approached with caution (e.g. by respecting the wishes of individuals from war-torn areas and ensuring their consent). Ongoing support and long-term plans should be in place to assist with residents’ readjustment upon their return home [33,34]. Given the absence of research bridging tourism and humanitarian crises, this paper presents a conceptual framework to identify obstacles and interdisciplinary approaches to promoting solidarity tourism for post-war recovery. By integrating knowledge from tourism, marketing, and public/global health, this viewpoint offers guidance for public health and tourism stakeholders (e.g. destination authorities, health care and tourism practitioners, and residents) to improve people’s health and well-being in war-torn societies.

## A CONCEPTUAL FRAMEWORK OF SOLIDARITY TOURISM, WAR, AND GLOBAL HEALTH

To facilitate post-war recovery, it is necessary to understand the long-term physical and psychological consequences potentially afflicting locals and tourists who visit affected areas. Disability, psychosocial illness, and travel safety are known problems threatening public health in war-affected societies. Solidarity tourism can play a role in alleviating conflict and supporting mental health. Considering the complexity of humanitarian crises in solidarity tourism, this study examines relevant challenges, implications, and marketing strategies for the post-war period.

First, it is difficult to collect and analyse data on people’s mental health, well-being, and other salient conditions in war zones. Scholars may thus face issues in disseminating accurate information to society to solicit support for these people during times of crisis. Interdisciplinary collaborations meant to encourage solidarity tourism can include big data analysis (e.g. refugee data collection and forecasting, clinical and public health information collection, social network computing) to better understand people in need during and after wars and to provide timely services, products, and experiences. The emergence of big data science has been intensively discussed in practical and academic fields. Researchers have sought to understand controversial, sensitive, and budding topics for which primary data are scarce or otherwise hard to obtain using methods such as surveys and interviews. Studies on war and tourism are largely descriptive and conceptual [35,36]. Social media and other internet channels (e.g. YouTube and Facebook) are ideal sources for big data analysis to uncover public perceptions of war or conflict and to learn about the mental health and emotional status of people living in war zones. Scholars should harness big data science to collect primary data and conduct relevant research, especially in times of crisis. Professional communication guidelines will also be required to filter misinformation and disinformation to ensure accurate decisions during turbulent periods.

**Figure:**
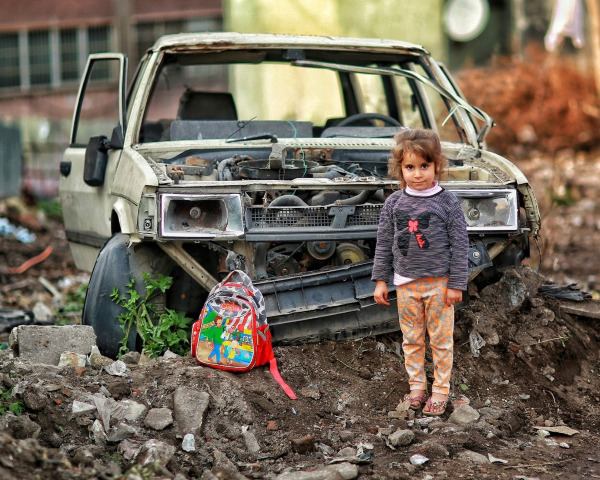
Photo: Girl with backpack near broken war ruins. Author: Berke Arakli. Source: Pexels, free to use (https://www.pexels.com/photo/girl-with-backpack-near-broken-car-ruins-8204704/).

Second, from a tourism marketing perspective, robust research is needed to understand how to address the needs of people in war zones. This gap presents an opportunity for interdisciplinary cooperation; cross-disciplinary scholars (e.g. in consumer psychology) would surely have valuable input. Related data can provide a solid foundation for solidarity tourism initiatives to shape evidence-based decisions. These individuals’ travel-related needs, wants, motivations, and expectations may be drastically different from other tourists when travel conditions are satisfied. Solidarity tourism stakeholders must carefully develop products and services with advice from medical specialists. This way, people who have experienced wars can benefit from tourism experiences centred around mental health and emotional healing. In addition, tourism brand images, destination images, cultural conflict, animosity, and other marketing factors [37-40] should be considered when assessing the unique characteristics of this niche tourist group. Salient marketing strategies should no longer solely focus on economic returns but should instead emphasise social responsibility within the tourism industry.

**Figure 1:**
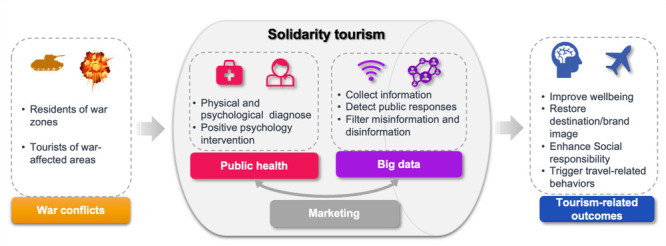
An infographic on solidarity tourism using an interdisciplinary approach.

Third, the COVID-19 pandemic highlighted the nexus between tourism and public health and provided evidence of tourism’s multifaceted role in people’s lives [41,42]. Regarding solidarity tourism, scholars in both this field and medicine should seize opportunities that can benefit people living in war zones when individuals consider travelling after war. Post-war consequences may include psychological and emotional disorders, physical injuries, and a sense of loss (e.g. of family or friends, a home, or the environment). Public health experts’ input can help the tourism industry in war-torn areas to rebound after the crisis. Many aspects of tourism can be productive for mental health, such as planning, anticipation, visiting, learning, and remembering [43]; these effects can be further tied to psychological benefits that accompany travel, such as reflecting on pleasant experiences, goal-directed thinking, and positive future thinking [44]. In sum, solidarity tourism initiatives should take advantage of multidisciplinary and industry collaborations to ensure that vulnerable groups can feel the empathy the tourism industry wants to convey as they heal from the effects of war. In the name of solidarity tourism after wars, there are some salient aspects that should provide insights to relevant stakeholders in the tourism field (**Figure 1**).

## CONCLUDING REMARKS

Although everyone presumably dreams of a world where peace prevails, war and conflict are sadly ongoing. We hope solidarity tourism will offer a practical solution to complex health emergencies and provide restorative experiences for those who have suffered through war. This viewpoint described several interdisciplinary approaches to support people in war zones via collaborations among tourism, marketing, and public/global health through the lens of solidarity tourism. First, this paper outlined the physical and psychological impacts of war-related conflicts on residents and tourists in war-torn societies. Building on the application of positive psychology in tourism research, public health scholars are urged to integrate a tourism perspective to explore how tourism could improve the health and well-being of war-affected individuals (e.g. children and refugees) through two pathways. Second, this article discussed challenges related to helping residents of war-torn societies. Big data technology can be applied to trace, predict, and understand groups in post-war research and practice. Further, we addressed the connections between public health, tourism, and marketing in post-war recovery. Beyond war, solidarity tourism can play a part in various crises, including natural disasters and pandemics. For example, it can contribute to the recovery of public health after the recent devastating Turkey-Syria earthquake. Empirical studies are recommended to continue elaborating on tourism’s role in resilience following humanitarian crises.
